# How Physical Factors Coordinate Virus Infection: A Perspective From Mechanobiology

**DOI:** 10.3389/fbioe.2021.764516

**Published:** 2021-10-29

**Authors:** Wei Liu, Daijiao Tang, Xin-Xin Xu, Yan-Jun Liu, Yaming Jiu

**Affiliations:** ^1^ Shanghai Key Laboratory of Medical Epigenetics, International Co-laboratory of Medical Epigenetics and Metabolism (Ministry of Science and Technology), Department of Systems Biology for Medicine, Zhongshan Hospital, Shanghai Institute of Cardiovascular Diseases, Institutes of Biomedical Sciences, Fudan University, Shanghai, China; ^2^ The Center for Microbes, Development and Health, Key Laboratory of Molecular Virology and Immunology, Institut Pasteur of Shanghai, Chinese Academy of Sciences, Shanghai, China; ^3^ University of Chinese Academy of Sciences, Beijing, China

**Keywords:** virus infection, mechanobiology, cytoskeleton, mechanosensors, shear stress, tensile or compressive forces, topography, organ-on-a-chip

## Abstract

Pandemics caused by viruses have threatened lives of thousands of people. Understanding the complicated process of viral infection provides significantly directive implication to epidemic prevention and control. Viral infection is a complex and diverse process, and substantial studies have been complemented in exploring the biochemical and molecular interactions between viruses and hosts. However, the physical microenvironment where infections implement is often less considered, and the role of mechanobiology in viral infection remains elusive. Mechanobiology focuses on sensation, transduction, and response to intracellular and extracellular physical factors by tissues, cells, and extracellular matrix. The intracellular cytoskeleton and mechanosensors have been proven to be extensively involved in the virus life cycle. Furthermore, innovative methods based on micro- and nanofabrication techniques are being utilized to control and modulate the physical and chemical cell microenvironment, and to explore how extracellular factors including stiffness, forces, and topography regulate viral infection. Our current review covers how physical factors in the microenvironment coordinate viral infection. Moreover, we will discuss how this knowledge can be harnessed in future research on cross-fields of mechanobiology and virology.

## Introduction

**FIGURE 1:**
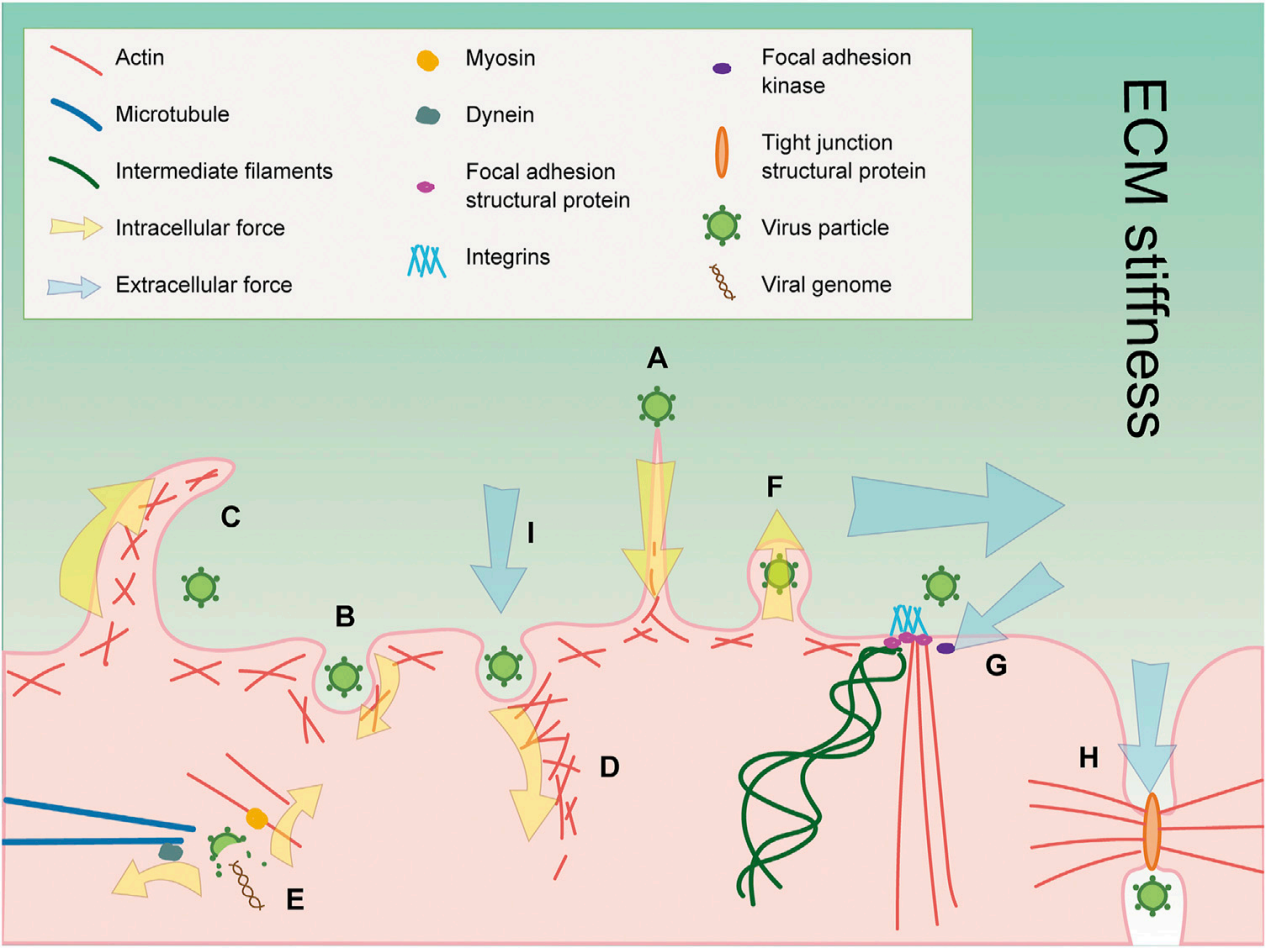
Cytoskeleton and mechanosensors play crucial roles during viral infections. **(A)** Actin filaments in host cells participate in virus surfing before entering into the cells. **(B)** Actin filaments provide forces for viral entry through clathrin-mediated endocytosis. **(C)** Macropinocytosis is employed by viruses for entry which is an actin-dependent process. **(D)** Actin monomers undergo rapid polymerization to generate forces for viral entry through caveolae-mediated endocytosis. **(E)** Microtubule and actin filament motor proteins dynein and myosin may provide forces for virus uncoating, respectively. **(F)** Actin filaments provide bending force to expel viruses to the extracellular environment. **(G)** Focal adhesion and FAK can sense extracellular mechanical signals such as shear force (horizonal arrow), tensile forces (slanting arrow), and ECM stiffness (gradient background color). Focal adhesion proteins can also affect viral infection in multiple ways. **(H)** Cell–cell junctions sense forces from intracellular and extracellular environments, and may be employed by viruses to facilitate their infection. **(I)** Caveolae sense extracellular stress. The number, morphology, and localization of caveolae are altered in response to stress and further affect viral infection.

**FIGURE 2:**
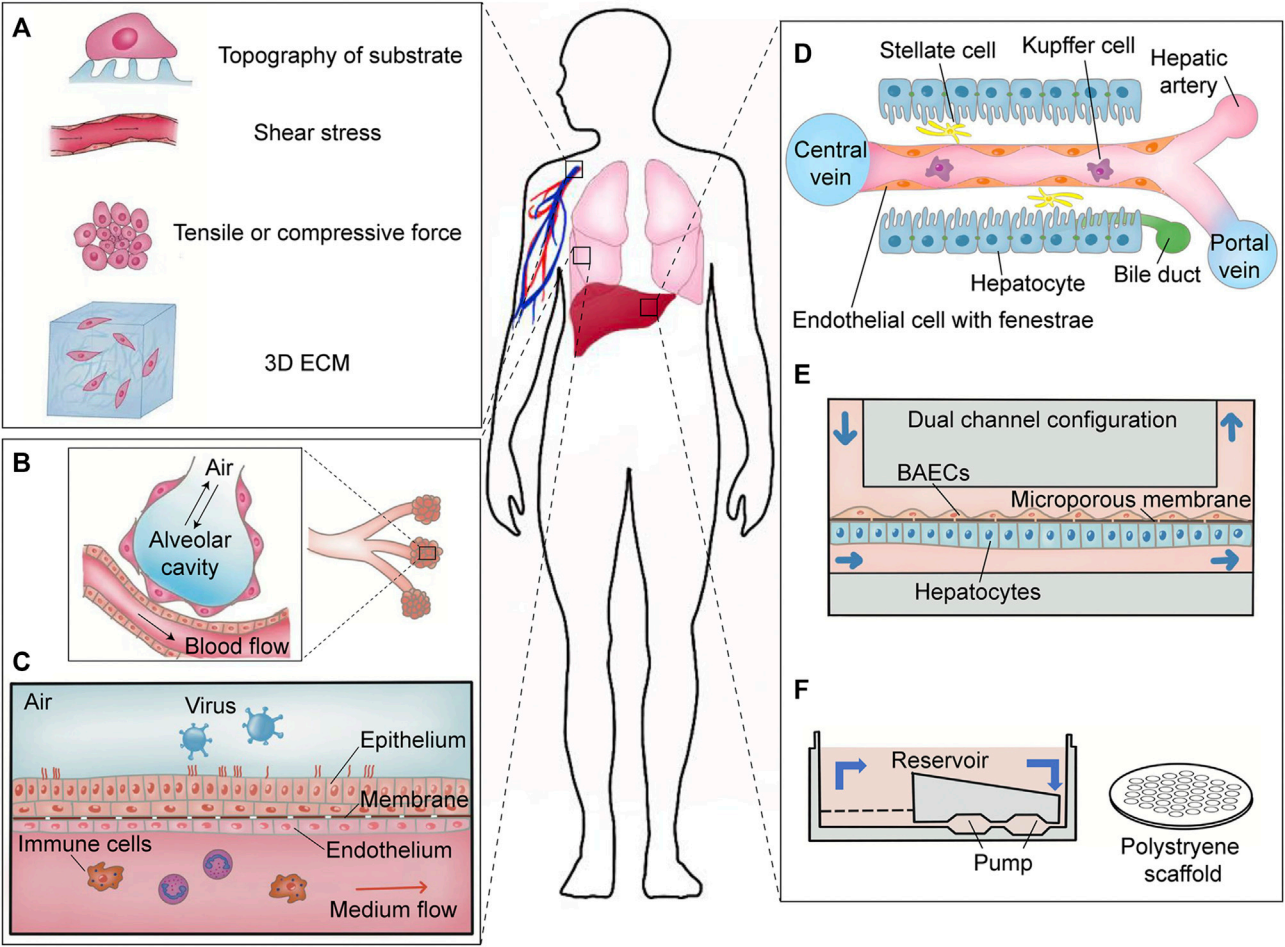
Extracellular mechanical forces *in vivo* and organ-on-a-chip models for virology *in vitro*. **(A)** Extracellular mechanical forces existing in the human body. Extrinsic physical parameters are ubiquitous *in vivo* like topography of the substrate formed by ECM, shear stress from fluid flow, tensile or compressive forces, and 3D ECM. **(B)** Physiological microenvironment in the human pulmonary alveoli. Shear force generated by blood flow and tensile force exerted on the alveolar cavity are important mechanical parameters for respiratory virus infection. **(C)** Schematic diagram of lung-on-a-chip. Adapted from ref. (Si et al., 2021). This biochip reconstituted the alveolar microenvironment including simulating blood flow and air exchange. **(D)** Physiological microenvironment in human liver sinusoid. Adapted from ref. (Ehrlich et al., 2019). Shear stress derived from biological flow is a crucial factor for maintaining the differentiation of hepatocytes *in vitro*. **(E)** Schematic diagram of a dual channel microdevice mimicking hepatic sinusoid. Adapted from ref. (Kang et al., 2015). **(F)** Schematic diagram of another liver-on-a-chip. Adapted from ref. (Ortega-Prieto et al., 2018).

Mechanobiology is a multidisciplinary research field ranging from biology to physics, and it focuses on the circulation of mechanosensation, mechanotransduction, and mechanoresponse (Howard et al., 2011; Krieg et al., 2019). With the in-depth research for complex mechanobiology, it has infiltrated, including biology, physics, mathematics, engineering, medicine, and biotechnology (Eyckmans et al., 2011; Polacheck et al., 2013; Schwarz, 2017; Naruse, 2018). Mechanical forces are ubiquitously exposed to cells, tissue, organs, and individuals, which directly or indirectly regulate their function. At the cellular level, the cytoskeleton including actin filaments, microtubules, and intermediate filaments constitute dynamic cytoskeletal structures with varied binding proteins, which sense and transmit extracellular mechanical loads or generate mechanical cues to the surrounding extracellular matrix (ECM) (Figure 1 and Figure 2) (Eyckmans et al., 2011). Specifically, integrins as transmembrane mechanoreceptors sensed biomechanical changes and transmitted forces to the cytoskeleton (Wang et al., 1993). During morphogenesis, biochemical factors like morphogens coupling with intrinsic and extrinsic mechanical cues were of vital importance in driving embryogenesis (Howard et al., 2011; Vining and Mooney, 2017). *In vivo*, cell behaviors are precisely regulated by multiple factors, including cell types, cell states, secretory proteins, and environmental information in the niche. In the microenvironments, there are various physical elements such as fluids, confined space, and topography apart from the biological and chemical context of cells. These forces individually or together exert mechanical cues to regulate cell behaviors (Figure 2A). Consequently, it is essential to develop advanced techniques to disentangle these physical elements and to study how individual mechanical cue affects cell behaviors. Attributing to the pioneering technologies, systems applied in mechanobiology have increasingly developed at a very fast pace and provide significant insights into mechanobiology.

Mechanobiology as an emerging research discipline has already extended to virology. Extrinsic and intrinsic mechanical forces can promote or impact virus infection. In this review, we focus on how to decouple each mechanical force *in vivo* and mimic the physical microenvironments *in vitro*, and elaborate how mechanical forces influence the process of viral invasion.

## Numerous Physical Parameters Affect Virus Infection

In cells, the cytoskeleton is the structure that is most intimately associated with cell mechanics. It functions in a lot of cell activities, including cell motility, cell morphology, intracellular transportation, cell division, force transmission, and endocytosis. There are three types of cytoskeletons: microtubules, actin filaments, and intermediate filaments (Figure 1) (Hohmann and Dehghani, 2019). Microtubules and actin filaments potentially provide forces for every step of virus life cycle, from entry to uncoating and from assembly to egress. Microtubule motors kinesin and dynein and their accessories such as the dynactin complex are responsible for force generation to drive cell activities like intracellular transportation and endocytosis (Goodson and Jonasson, 2018). Actin motors such as myosin and actin polymerization factors like the Arp2/3 complex are critical for force generation by actin filaments (Blanchoin et al., 2014). Intermediate filaments can be divided into six types, and each is formed by different kinds of proteins. Intermediate filaments are significant for cells to resist stress and, together with microtubules and actin filaments, could sense extracellular mechanical signals by associating with mechanosensors and activate downstream signaling pathways (Fletcher and Mullins, 2010; Goldmann, 2018), for example, the FAK-Src signaling pathway, which is vital for cell migration (Cary et al., 1996), and the Hippo pathway as well as YAP/TAZ, which regulates cell proliferation, survival, and maintenance in response to mechanical cues (Dasgupta and McCollum, 2019). Many viral infections are dependent on mechanosensors and their protein components, thus providing another way for the cytoskeleton to mechanically regulate viral infections (Gregor et al., 2014).

Extracellular mechanical signals are transmitted into cells by different mechanosensors and lead to a series of cell mechanic changes. In the next section, we will focus on three kinds of mechanosensors: focal adhesion, cell–cell junction, and caveolae, to introduce their roles during viral infection (Figure 1). Focal adhesion is large adhesion contacts at the ends of actin stress fibers (Petit and Thiery, 2000). Its structure protein integrin and regulatory protein focal adhesion kinase (FAK) are able to sense mechanical signals including tensile forces, shear stress, and extracellular matrix stiffness. Meanwhile, focal adhesion is employed by a lot of viruses to facilitate their infection. Cell–cell junctions link cells to each other in tissues. There are three types of cell–cell junctions, including tight junctions, adherent junctions, and desmosomes (Garcia et al., 2018). Cell–cell junctions can sense forces from intracellular and extracellular environments and transduce the mechanical signals to promote cell adaptions to the environment (Yap et al., 2018). Cell–cell junctions are also involved in viral infections and affect the viral infections in various ways. Caveolae are rounded invaginations on the plasma membrane, and caveolin-1 and cavin-1 are indispensable structural proteins of it. Extracellular stress is proved to be sensed by the caveolae and could alter its number, morphology, and localization (Sinha et al., 2011). Since many viral infections are dependent on caveolae (Xing et al., 2020), caveolae may bridge the interactions between viral infections and stress.

Extracellular mechanical factors during viral infections are divided into four types: shear stress, tensile or compressive forces, 3D ECM, and topography of substrates (Figure 2A). *In vivo*, bloodstream flowing above epithelial cells generates shear stress on cells and alters their formation and function (Souilhol et al., 2020). Extracellular shear stress has been confirmed to influence viral infections in different *in vitro* models. Tensile or compressive forces, sensed by the cytoskeleton and mechanosensors, may affect the viral infection in multiple aspects. Organ-on-a-chip models have been employed to study the viral infections in the presence of mechanical forces (Villenave et al., 2017). Tissue stiffness could be changed by noninfectious (Schutte et al., 2020) and infectious factors such as viral infections (Vinikoor et al., 2016). It could be sensed by focal adhesions and results in extensive cell mechanical changes, which might affect different steps in viral infections. Different tissues have various topographies, which may influence cell mechanics and motility, thus affecting the viral infection and transmission (Xu et al., 2014).

## Host Cytoskeleton and Mechanosensors During Viral Infection

The cytoskeleton and mechanosensors are significant intracellular physical factors that may alter cell mechanics and influence viral infections (Figure 1). The cytoskeleton is responsible for generating forces for various cell activities. During viral infection, the forces generated by the cytoskeleton might be utilized by viruses to facilitate their infections. The cytoskeleton can also sense mechanical cues by associating with mechanosensors (Fletcher and Mullins, 2010). Mechanosensors are cell structures and proteins that are able to sense different kinds of extracellular mechanical signals and transduce them to intracellular to activate downstream signaling pathways and lead to cell mechanic changes. These changes may affect viral infections indirectly in multiple ways. Besides, mechanosensors might be employed by viruses to facilitate their infection. Therefore, how mechanosensors regulate viral infection mechanically may be a potential research field and lead to the discovery of new antiviral targets.

### How Cytoskeleton Mechanically Regulates Viral Infection

Actin cytoskeleton and microtubules, with their associated proteins, are able to respond to a variety of mechanical signals and generate physical forces for plenty of cell activities, such as intracellular cargo transportation and cell motility (Fletcher and Mullins, 2010). Intermediate filaments are well known to provide mechanical support against stress (Goldmann, 2018). Importantly, the cytoskeleton is extensively involved in viral infections, functioning as transporters of viral particles, physical barriers to resist viral entry, *etc* (Goldmann, 2018). Therefore, the mechanical properties of the cytoskeleton should be a significant factor required to be considered in virology studies. In this part, we summarize recent studies about how the host cytoskeleton affects viral infection through mechanical regulation and advanced insights of this research field.

After binding to certain receptors on the plasma membrane, it is necessary for viruses to migrate to preferred sites for entry. The most common form of this process is virus surfing, an actin-dependent movement of the virus toward the cell body. The underlying mechanism revealed that actomyosin generates forces for retrograde flow and subsequently pulls the filopodia-associated actin filaments towards the cell body (Spear and Wu, 2014). It had been proved that the entry of murine leukemia virus (MLV) (Lehmann et al., 2005) and herpes simplex virus (HSV) utilized virus surfing in an actin-dependent manner (Salameh, 2012).

The majority of viruses enter cells through endocytosis (Mercer et al., 2010, 2020), which is further divided into clathrin-mediated endocytosis and caveolae-mediated endocytosis. The actin cytoskeleton and microtubules are indispensable for endocytosis due to their force-generating ability. Arp2/3 complex, myosin, and other actin-related proteins together control and regulate the polymerization and growth of the actin network and provide force to generate the invagination of the membrane in clathrin-mediated endocytosis (Kaksonen et al., 2006). Rhabdoviruses entered cells through the clathrin-mediated pathway in an actin-dependent manner: cytochalasin D treatment impeded viral entry (Guo et al., 2019). Adenoviruses entry was through clathrin-mediated and actin- and dynein-dependent endocytosis (Meier and Greber, 2004). Mosquito-borne flaviviruses, such as Japanese encephalitis virus (JEV) and West Nile virus (WNV), typically entered cells through the clathrin-mediated pathway. The disruption of actin filaments using cytochalasin D and jasplakinolide inhibited JEV entry (Kalia et al., 2013), and the disruption of the microtubule network by nocodazole strongly affected the WNV entry (Chu et al., 2006). Actin dynamics are also necessary for Kaposi’s sarcoma–associated herpesvirus (KSHV) entry through clathrin-mediated endocytosis since disruption of the actin cytoskeleton and inhibition of regulators of actin nucleation blocked KSHV entry and trafficking (Greene and Gao, 2009).

Macropinocytosis is even more tightly associated with actin, since it is an actin-driven process. Actin polymerizes in a ring under the cell membrane to form the macropinocytic cup, and myosin provides contractile force for the cup to close and seal (King and Kay, 2019). KSHV entered human dermal microvascular endothelial (HMVEC-d) cells predominantly through macropinocytosis, and the infection induced myosin light chain II phosphorylation. Myosin might provide forces to produce the movement requested by the process of bleb retraction (Veetti et al., 2010). Knockdown of TSPAN7, a regulator of actin nucleation, led to increased macropinocytosis of human immunodeficiency virus 1 (HIV-1) in dendritic cells while the inhibition of actomyosin contraction was able to rescue the knockdown (Ménager, 2017). Hantaan virus (HTNV) and Andes orthohantavirus (ANDV) entered human respiratory epithelial cells probably through macropinocytosis since their entry depended on sodium proton exchangers and actin (Torriani et al., 2019).

Actin polymerization is also essential for the formation and budding of caveolae (Hinze and Boucrot, 2018). Simian virus 40 (SV40) is well known for employing the caveolae-mediated endocytosis pathway for entry. Specifically, SV40 triggered a signal transduction cascade which led to depolymerization of the actin filaments under plasma membranes. Generated actin monomers were then recruited to the virus-loaded caveolae and formed an actin patch, on which a burst of actin polymerization occurred. Virus-loaded caveolae vesicles were subsequently released from the membrane and moved into the cytoplasm (Pelkmans and Helenius, 2002). Before transmissible gastroenteritis virus (TGEV) internalization, caveolin-1 would gather around the viruses with the assistance of actin and clathrin to form the vesicle containing TGEV, and after ∼60 s, dynamin 2 was recruited to promote membrane fission (Wang C. et al., 2020).

Apart from endocytosis, the cytoskeleton network also functions in uncoating, replication, and assembly steps during the viral life cycle. Microtubule- and actin-associated motors, including dynactin, dynein, and myosin II, generated physical forces to help break apart capsids of the influenza A virus (IAV) and thus promoted its entry (Banerjee et al., 2014). Cytoskeleton rearrangement and dynamic changes are common phenomena among a lot of viruses’ replications, such as coronavirus (Wen et al., 2021) and HIV-1 (Spear et al., 2014) and may mechanically affect viral replication since cytoskeleton rearrangements always lead to extensive cell mechanic changes (Spear and Wu, 2014). Cytoplasmic forces also contributed to vaccinia viral replication by translocating the replication sites towards the nucleus (Schramm et al., 2006). As for assembly, it was theoretically assumed that actin filaments provide protrusive forces to initiate assembly during retroviral infection (Gladnikoff et al., 2009).

In addition, the cytoskeleton is indispensable for the egress of viruses. Actin nucleation might offer driving force to expel the virus from membrane pits to the extracellular environment (Newsome and Marzook, 2015). Release of vaccinia virus (VACV) required the force of actin nucleation to reduce the association between the extracellular virus and plasma membrane (Horsington et al., 2013). During measles virus (MV) budding, the actin cytoskeleton performed a vectorial growth which might generate forces contributing to the formation of viral buds (Bohn et al., 1986). Intact actin cytoskeleton was crucial in providing force necessary to expel WNV to the extracellular environment (Chu et al., 2003). In SARS coronavirus–infected cells, actin filaments which were parallel to the cell edge might thicken to provide bending force to expel viral particles from the plasma membrane (Ng et al., 2004).

In addition to affecting different steps in the viral life cycle, the actin cytoskeleton regulates viral infection by altering signaling pathways. The Rho–ROCK–Myosin II contractility signaling pathway increased cell stiffness and formed a physical barrier against viral infection (Das et al., 2015). Decreased actin polymerization led to the translocation of the NF-κB transcription factor p65 to the nucleus, and the NF-κB signaling pathway was known to have antiviral function (Uhler and Shivashankar, 2017). The cytoskeleton is very closely related to cell mechanics that can alter a great amount of cell activities and affect viral infection indirectly.

Different types of intermediate filaments locate at different sites and execute distinguished functions. Although they do not have motor proteins like actin filaments and microtubules do, they are indispensable for cells to resist stress and are involved in the mechanosensing of cells. Therefore, they are vital for a variety of cell activities including cell migration, mitosis, cell growth, and stress-mediated responses (Sanghvi-Shah and Weber, 2017). Keratin adapts to different matrix rigidities, regulates stiffness-dependent F-actin remodeling, and transduces the mechanical signals to the nucleus lamina (Laly et al., 2021). Focal adhesion–anchored vimentin could regulate mechanosensing by activating FAK and its downstream signaling pathways (Gregor et al., 2014). Intermediate filaments also affect viral infection in multiple aspects. Cell surface vimentin functioned as a coreceptor to help the SARS–CoV spike protein bind to receptor angiotensin-converting enzyme 2 (ACE2) (Yu et al., 2016). For human papillomavirus 16 pseudovirions (HPV16-PsVs), knocking down of cell surface vimentin with siRNA significantly increased its binding and internalization (Schäfer et al., 2017). Vimentin was also critical for IAV genome penetration into the cytoplasm to facilitate viral infection. Vimentin depletion severely reduced IAV RNA, protein expression, and production of infectious viral particles (Wu and Panté, 2016).

### Mechanosensors

Numerous cell structures and molecules are able to sense and respond to extracellular mechanical signals. Among them, focal adhesion, cell–cell junction, and caveolae are extensively studied and intimately associated with viral infection (Figure 1) although few studies have explored the relationship between these mechanosensors and viral infections from the perspective of mechanobiology. We summarize how these mechanosensors sense mechanical cues and affect diverse steps directly or indirectly during a viral infection.

Focal adhesion is a specialized region on the plasma membrane at which actin bundles are anchored to the integrin transmembrane receptors through a multimolecular complex of junctional plaque proteins (Petit and Thiery, 2000). Integrin interacts with extracellular matrix proteins to sense shear stress and activates downstream signaling molecules in focal adhesions and cytoplasm (Shyy and Chien, 2002). FAK is a well-known mechanosensor which is activated by tensile forces transmitted from cytoskeleton-anchored focal adhesion targeting (FAT) domain and membrane through the phosphoinositide phosphatidylinsositol-4,5-bis-phosphate (PtdIns (4,5) P2) binding site (Zhou et al., 2015). Focal adhesion and related proteins are also involved in viral infections in numerous aspects. For instance, FAK regulates the phosphorylation and transcriptional activity of NF-κB in response to fluid shear stress (Petzold et al., 2009). Porcine hemagglutinating encephalomyelitis virus (PHEV) caused an actin filament rearrangement through the integrin α5β1-FAK-Rac1/Cdc42-PAK-LIMK-cofilin pathway to facilitate its own infection (Lv et al., 2018). IAV hijacked FAK to promote its replication and inhibited FAK from activating innate immune responses (Bergmann and Elbahesh, 2019). Integrin was employed by a variety of viruses as a cellular receptor or internalization factor, such as WNV (Bogachek et al., 2010), zika virus (ZIKV) (Wang S. et al., 2020), adeno-associated virus (AAV) (Summerford et al., 1999), and adenovirus (Lyle and McCormick, 2010) to promote their infection.

Cell–cell junctions connect cells with each other and regulate tissue homeostasis during tissue barrier homeostasis, cell proliferation, and migration. They also function in mechanosensing and mechanotransduction of forces from multiple sources, such as external forces applied at the tissue scale, forces generated within tissues, and cellular contractility (Yap et al., 2018). Tight junction, a type of cell–cell junction, usually serves as physical barriers to resist pathogens invasion. However, some viruses may interact with tight junction–related proteins to promote their entry. The best-studied case is that adenovirus bound to coxsackievirus and adenovirus receptor (CAR), a tight junction integral protein to cross the human airway epithelial layer and entered cells for replication (Walters et al., 2002). Claudin-1, another tight junction protein, was a hepatitis C virus (HCV) coreceptor required for its entry (Evans et al., 2007). In addition to tight junction–associated proteins, the adherent junction protein nectin-4 served as an epithelial receptor for MV (Mühlebach et al., 2011).

Caveolae have been confirmed to undergo assembly and disassembly as well as localization and morphology change in response to mechanical stress (Boyd et al., 2003; Sinha et al., 2011). Caveolin-1 (Cav-1), a critical protein component of caveolae, is significant in regulating actin-related mechanosensitive pathways (Echarri and Del Pozo, 2015). Meanwhile, caveolae not only regulate viral entry but also other steps in the viral life cycle. Cav-1 bound to the HIV Env protein at the caveolae lipid raft, and the interaction blocked HIV fusion and reduced viral replication (Wang et al., 2010). Respiratory syncytial virus (RSV) morphogenesis proceeded within caveolae, and both Cav-1 and cavin-1, two major components of caveolae, were recruited to and incorporated into the RSV envelope, which occurred just before the RSV filament assembly (Ludwig et al., 2017). Paramyxovirus parainfluenza virus 5 (PIV-5) virions lacking Cav-1 were defective and contained high levels of host proteins and low levels of viral hemagglutinin-neuraminidase (HN) and matrix (M) proteins, suggesting that Cav-1 was incorporated in mature PIV-5 particles. Besides, Cav-1 was clustered at sites of PIV-5 budding (Ravid et al., 2010). Human parainfluenza virus type 2 (hPIV-2) V protein bound to and stabilized cavin-3, which in turn promoted assembly and budding of hPIV-2 in lipid raft microdomains (Ohta et al., 2020).

### Mechanobiology in Coronavirus

Regarding the recent pandemic of COVID-19, it is urgent to uncover the mechanisms of coronavirus infection to provide more possible targets for precaution and therapy. A perspective of mechanobiology may offer some new understandings to coronavirus, whose life cycle is intimately associated with and regulated by different kinds of forces. Generally, the cytoskeleton generates forces and may broadly affect cell mechanics to mechanically regulate coronavirus infection. Human coronavirus NL63 (HCoV-NL63) (Milewska et al., 2018) requires dynamic actin cytoskeleton for their replication and release. As mentioned in the previous part, SARS-CoV infection resulted in thickened actin filaments below the subcellular surface, which may provide bending force to expel the virus particles (Ng et al., 2004).

Except for traditional virology and cell biology technologies, plenty of mechanobiology technologies have been employed to shed light on mechanics involved in coronavirus infection. A recent study reveals that tensile force, generated by bending of the host cell membrane, enhances the recognition of SARS–CoV-2 spike with ACE2 to facilitate the detachment of S1 from the S2 subunit to initiate the viral fusion machinery (Hu et al., 2021). Shear stress caused by the risk factors hypertension may induce post-translational modifications of host cell proteins mimicked by SARS-CoV-2 proteins and further lead to the change of plasma-cell membrane localization and autoimmune-induced endothelial damage (Gammazza et al., 2020). Moreover, organ-on-chips have been used to study effects of coronavirus infection on different organs, such as the gut (Kaneko et al., 2021) and lung (Zhang et al., 2021), and accelerate the identification of therapeutics and prophylactics with potential (Si et al., 2021). Mechanobiology technologies allow us to customize a specific simulative environment or mechanical state to study coronavirus infection under certain physical conditions, which is more precise and controllable than traditional *in vitro* or *in vivo* techniques.

## Extracellular Mechanical Forces During Viral Infection

There are various culture systems *in vitro* that have been applied in virology, aiming at elucidating the pathogenesis of virus infection, host–virus interactions and host immune responses, and dedicating to drug discovery and vaccine development. Most of these culture systems are built on 2D multiwell plates in which cells are seeded on plastic or glass bottom during a viral infection. However, it is much more sophisticated pathophysiology for host–virus interactions *in vivo* (Vining and Mooney, 2017). For example, vascular endothelial cells are exposed to shear forces of blood stream rather than in static culture condition. One of the disadvantages of conventional models of virus infection is that these systems cannot accurately simulate the real microenvironment of viral infection. Microfabrication and nanofabrication technology have been rapidly evolved in the recent years and has established novel approaches to study how mechanical forces influence virus infection *in vitro*, which can better mimic the microenvironment *in vivo* (Tang et al., 2020). Here, important extrinsic mechanical forces *in vivo* during virus infection are divided into three types: shear stress, tensile or compressive forces, and topography of the substrate (Figure 2A). We summarize the extrinsic mechanical forces that affect kinetics and pathogenesis of a viral infection and discuss how these physical factors can be applied in the future antiviral studies.

### Shear Stress

Shear stress, a frictional force generated by the blood stream, exerts mechanical stimulus on endothelial cells that affect its function (Traub and Berk, 1998; Souilhol et al., 2020). During early embryo development, it is vital for fluid shear stress to adjust and control left–right body asymmetry (Vining and Mooney, 2017). Under realistic physiological conditions, biological fluids serve as naturally physical barriers to hold back adsorption and invasion of the causative agent, and therefore, pathogens have developed exquisite strategies to break through physical shear forces in the body (Tonkin and Boulanger, 2015).

Shear-flow turbulence at some specific sites in blood vessels can function as mechanical cues to activate latently herpes virus–infected endothelial cells (Jacob, 1994). This kind of activation changed the expression of heparans on the cell surface, which was one of the causative factors inducing atherogenesis. Similarly, exposure to low shear stress which mimicked the mechanical microenvironment of atheroprone regions *in vivo* promoted the infection of human cytomegalovirus (HCMV) to endothelial cells (DuRose et al., 2012). However, there were little significant differences for HCMV infection when endothelial cells were under high shear stress or in static conditions. Shear forces applied to endothelial cells would alter the gene expression. Comparing brain endothelial cells cultivated in conventional 2D- and 3D-printed vascular model, shear flow in the 3D model increased the expression of angiotensin-converting enzyme-2 (ACE2) resulting in severe acute respiratory syndrome coronavirus-2 (SARS-CoV-2) infection (Kaneko et al., 2021). Dynamic culture system in a microchannel provided NIH/3T3 cells with more susceptible condition for virus infection in contrast with the conventional petri dish culture (Kim et al., 2018). These studies demonstrated that shear forces from blood flow is a crucial mechanical stimulus affecting viral infection. *In vitro*, propagation and production of virus models also confirmed that the flowing shear stress influenced the viral infection. A suitable shear stress below 0.25 N m^−2^ would enhance the titers of the oncolytic measles virus in the viral propagation model (Grein et al., 2019). Hydrodynamic shear forces generated from an agitated bioreactor increased propagation of JEV in Vero cells (Wu and Huang, 2000). Notably, the value of shear stress can be different in different culture systems as an agitator-dependent shear over 0.25 N m^−2^ would decrease the titer of the oncolytic measles virus (Grein et al., 2019), and shear stress origin from gas bubbles was harmful for a baculovirus-expressed vector system (Weidner et al., 2017). It was proposed that increased endothelial pulsatile shear stress can be a good choice to prevent SARS-CoV-2 infection by increasing bioavailability of nitric oxide (NO) (Sackner and Adams, 2020). Different classes of cells exposed to the same value of shear forces also showed different performances during viral infection. Compared with BHK-21 cells, Vero cells in a microcarrier were more vulnerable to JEV (Wu and Huang, 2000). Viral invasion can cause cytopathogenic effect (CPE) in individual cells. Interestingly, vaccinia virus, a member of poxvirus, promoted cell migration which was one of the distinctive CPE (Sanderson et al., 1998). It was also found that there is enhanced directional cell migration induced by VACV in the presence of shear stress in a microfluidic device (Wang et al., 2017). It was because that the fluid flow reduced extra lamellipodium around the infected cell and changed the orientation of the Golgi complex.

In addition, emergence of human organ-on-a-chip offers new insights into investigation of the mechanisms of virus–host interactions (Tang et al., 2020). To improve conventional viral models and better simulate real microenvironment *in vivo*, shear stress as a significant mechanical cue is usually introduced into organ-on-a-chip (Figures 2C,E,F). It was proved that the recirculation of culture media was helpful to recapitulate the complex hepatic sinusoid *in vitro,* and this 3D microfluidic model can be applied to study the dynamics and mechanism of hepatitis B virus (HBV) infection (Ortega-Prieto et al., 2018). Shear forces were also applied to the distal renal tubules model to explore the association between renal dysfunctions and viral infections (Wang et al., 2019b).

### Tensile or Compressive Forces

External forces like tensile or compressive forces play a significant role in tissue morphogenesis. Mitotic spindle orientation can be modulated by applied stretch forces, which was associated with the location of cortical actin (Fink et al., 2011). During metastasis events, the tumor cells adjusted themselves to mechanical cues (such as ECM stiffness, compressive stress, and shear stress) of the microenvironment for their survival (Chaudhuri et al., 2018). Importantly, this type of force also affects viral infection from multiple aspects. Enteroviruses, a type of nonenveloped, single-stranded RNA viruses, primarily infect the gastrointestinal epithelial cells, contributing to the occurrence of many diseases, including exanthemas and poliomyelitis (Racaniello, 2006). Due to the complicated microstructure of the human intestinal epithelium, it is too simplified to use monolayer cells *in vitro* as an infection model to study enteric virus biology. Cyclic suction designed for exerting tension and compression force was used in a human gut-on-a-chip in order to mimic gastrointestinal peristalsis (Villenave et al., 2017). This device displayed excellent performance for villus-like structure formation and coxsackievirus B1 (CVB1) infection. The model showed that virus particles and inflammatory cytokines were detected at the cell apex, indicating that the mechanical forces were essential elements of the recapitulating complex intestinal epithelial microenvironment.

### Topography of the Substrate

Micro/nanostructured topographies of ECM pose a great diversity of mechanical cues to the cells or tissues surrounding them. Contact guidance as a way of cell responses to topographies is a general phenomenon during cell migration *in vivo* (Bettinger et al., 2009). Fibroblasts exhibited different forms of morphologies in responding to different topographies of the substrate (Ghibaudo et al., 2009). Topography also influences viral infection and transmission. Vero cells seeded on a microgrooved substrate showed anisotropic cell-to-cell transmission of VACV compared with those on a smooth substrate (Xu et al., 2014). The cytoskeleton rearrangement played a major role in cellular response to the microgrooved substrate that accounted for this redirection of cell-to-cell viral spread. As mentioned above, VACV infection promotes epithelial cell migration to speed up the spread of the virus. Topographic microstructures acting as contact guidance facilitated directed cell motility induced by VACV (Wang et al., 2019a). Reorientation of the Golgi complex and a dominant elongated protrusion was responsible for this directed cell migration.

### Organ-on-a-Chip

Although different mechanical parameters have been individually investigated during viral infections, it is not sufficient to thoroughly understand the interplay between dynamic physiochemical microenvironments and infectious viral particles. Advent of organ-on-a-chip technologies provides novel insights into exploring how spatial information regulate virus infection, which recapitulate the sophisticated microarchitecture of localized tissue and dynamic physiochemical microenvironments.

Advanced lung chip mimicking alveolar-capillary interface of the human body (Figure 2B) reconstituted an ingenious microdevice to offer an alternative model for drug discovery and preclinical trials (Huh et al., 2010). Mechanical cues like shear stress, tensile or compressive forces, and 3D coculture were integrated in this microsystem to achieve an organ-level lung chip. Using this lung chip, more detailed information and new phenomena during influenza virus and SARS-CoV-2 infection can be achieved, and cytokine M-CSF may be identified as a candidate marker indicating chronic obstructive pulmonary disease (COPD) caused by respiratory viruses (Benam et al., 2016; Si et al., 2019). In another lung chip, NCI-H441 cells and human bronchial epithelial cells were cocultured with monocyte-derived macrophages at the interface of a porous membrane that further resembled the cellular component of the human alveolus (Deinhardt-Emmer et al., 2020). Shear forces created by peristaltic pumps and a coculture of circulating immune cells increased barrier integrity formed in this biochip (Zhang et al., 2021). Coinfection of the influenza virus and *Staphylococcus aureus* destroyed the vascular endothelial barrier rather than the alveolar epithelial barrier, showing that pathogen infections can cause multi-impacts on the alveoli of the lungs. The same type of biochip was constructed to identify key features of human rhinovirus strain 16 (HRV-16)-induced exacerbation of asthma (Nawroth et al., 2020).

The hepatic sinusoid universally found in the liver is a kind of special capillary, which is regarded as a functional unit of liver activity (Figure 2D). A liver sinusoid provides a venue for mixing oxygen-rich arterial blood and nutrient-rich venous blood and also serves as a portal of entry for hepatitis virus (Wohlleber and Knolle, 2016). To date, few models *in vitro* mimicking the hepatic sinusoid are available due to the complex components of a sinusoid and dedifferentiation of the primary human hepatocyte cultured *in vitro* (Nahmias et al., 2007; Chen et al., 2012; Rowe et al., 2013). Liver-on-a-chip can offer a feasible solution. A dual-channel chip was separated by a porous membrane simulating the space of Disse between sinusoidal endothelial cells and hepatocytes. Primary rat hepatocytes or primary human hepatocytes and immortalized bovine aortic endothelial cells were cultivated on the opposite surface of the membrane with a continuous perfusion device mimicking shear stress from fluid flow (Figure 2E) (Kang et al., 2015, 2017). Under the condition of combined mechanical forces, primary hepatocytes in the chip can maintain their polygonal morphology for more than 3 weeks. Recombinant adenoviruses encoding the genome of HBV or isolated HBV from HepG2.215 or HepAD38 cell culture were able to infect hepatocytes in microchannel and accomplish HBV replication that verified the practicability of this kind of liver chip. Furthermore, a more simplified 3D microfluidic liver chip was developed to study HBV and screening of new anti-HBV drugs. This configuration with the recirculation of culture media used collagen-coated polystyrene scaffold as the substrate supporting primary human hepatocytes (Figure 2F) (Ortega-Prieto et al., 2018, 2019). This platform only containing the scaffold and circulatory system was much simpler than the microsystems described above. Primary human hepatocyte alone or cocultured with primary Kupffer cells retained viability and a dedifferentiated phenotype in this device for up to 40 days. Not only HepDE19-derived HBV at a low MOI = 0.05 genome equivalents (GE)/cell were able to infect 3D hepatocytes but also patient-derived HBV at a high MOI = 100 GE/cell. The secretion of cytokines (IL-8, macrophage-inflammatory protein (MIP)-3α, SerpinE1, and monocyte chemotactic protein-1 (MCP-1)) was similar to the test results from the sera of HBV-infected patients. This 3D microfluidic liver chip showed great potential in the application of anti-HBV therapy.

Gastrointestinal mucosae initially interact with enterovirus and are considered as an ideal architecture exploring the host–pathogen interplay. These tissues are constituted by multicomplex elements such as numerous cell types, 3D tissue architecture, and intestinal gurgling (Barrila et al., 2018). However, enterovirus models *in vitro* mostly build on single-type cell cultures forming flat monolayers, which lack precise regulation of the dynamic microenvironments. A human gut-on-a-chip explored how dynamic mechanical forces influenced intestinal function (Kim et al., 2016). This microengineered device comprised three parallel microchannels fabricated by poly (dimethylsiloxane) (PDMS). The central channel was separated by the ECM-coated PDMS membrane and the two-sided channels were drove by cyclic suction to generate cyclic peristalsis–like mechanical deformations. When ceasing tensile and compressive forces that exerted on human intestinal epithelial cells by stopping cyclic suction and remaining fluidic flow, the growth of enteric microorganisms was promoted, which meant mechanical cues influenced interactions of the host and pathogen. This microengineered model was further improved to apply in the CVB1-infected model (Villenave et al., 2017). Caco-2 intestinal epithelial cells cultured in this gut-on-a-chip displayed villus-like structures under conditions of continuous perfusion and cyclic mechanical strain. In this chip, viral particles and cytokines induced by CVB1 tend to be released from the apex, implying the polarized infection of CVB1 in gastrointestinal microenvironments.

Similar construction utilizing the porous membrane played a role in the kidney-on-a-chip to study virus-related renal dysfunctions (Wang et al., 2019b). Madin–Darby canine kidney (MDCK) cells were cultured on the upper surface of the porous membrane and exposed to microfluidic flow mimicking shear force from tubular flow distal renal tubules. Distal tubule-on-a-chip (DTC) combining shear stress with confined force provided epithelial cells a suitable physical microenvironment to form self-assembled microvilli. During pseudorabies virus infection, the disordered function of Na^+^ reabsorption and intertwined microvilli in DTC were observed, which opened new perspectives of dynamic changes after virus infection. Recently, an Ebola virus model built on a microvessel-on-a-chip permitted mechanistic studies of the Ebola hemorrhagic syndrome. The study showed that the Ebola glycoprotein (GP 1, 2) hijacked the Rho/ROCK pathway and modulated the host cytoskeleton resulting in albumin leakage from the biomimetic vascular wall (Junaid et al., 2020). It was worth noting that Ebola VLPs did not contain viral genome, and this phenotype was induced only by the glycoprotein on the surface of the virus.

## Conclusions and Perspectives

In summary, we highlighted the types of viruses, mechanical forces for its infection and transmission, major cellular components, and processes facilitating infection (Table 1).

**TABLE 1:** Mechanical forces regulate viral infection and transmission.

Type of virus	Intracellular and extracellular mechanical forces	Major cellular components/processes	References
Murine leukemia virus (MLV)	Actin filaments regulated retraction force	Virus surfing for entry	Lehmann et al. (2005)
Herpes simplex virus (HSV)	Actin filaments regulated retraction force	Virus surfing for entry	Salameh (2012)
Rhabdovirus (RV)	Actin filaments regulated contractile force	Providing force for clathrin-mediated endocytosis	Guo et al. (2019)
Adenovirus (AdV)	Actin filaments regulated contractile force	Actin and dynein providing forces for clathrin-mediated endocytosis; integrin as a receptor of the virus; Coxsackievirus and adenovirus receptor binding to the virus to facilitate its entry	Meier and Greber (2004)
			Lyle and McCormick (2010)
			Walters et al. (2002)
Kaposi’s sarcoma–associated herpesvirus (KSHV)	Actin filaments regulated contractile force	Providing force for clathrin-mediated endocytosis, macropinocytosis, and trafficking	Greene and Gao (2009)
			Torriani et al. (2019)
Simian virus 40 (SV40)	Actin filaments regulated contractile force	Providing force for caveolae-mediated endocytosis	Pelkmans and Helenius (2002)
Transmissible gastroenteritis virus (TGEV)	Actin filaments regulated contractile force	Providing force to promote membrane fission	Wang et al. (2020a)
Measles virus (MV)	Actin filaments regulated contractile force	Actin filaments generating forces for the formation of viral buds; adherent junction protein nectin-4 as a receptor for the virus	Bohn et al. (1986)
			Mühlebach et al. (2011)
Porcine hemagglutinating encephalomyelitis virus (PHEV)	Actin filaments regulated contractile force	Actin filaments rearrangement through the FAK-participated pathway to facilitate infection	Lv et al. (2018)
Retrovirus (RV)	Actin filaments regulated protrusive force	Providing protrusive forces to initiate assembly	Gladnikoff et al. (2009)
SARS coronavirus (SARS-CoV)	Actin filaments regulated bending force	Providing bending force to expel viral particles from the plasma membrane	Ng et al. (2004)
West Nile virus (WNV)	Microtubules regulated contractile force; Actin filaments regulated contractile force	Microtubules providing force for clathrin-mediated endocytosis; Actin filaments providing force to expel viral particles to the extracellular environment; Integrin as a putative receptor of the virus	Chu et al. (2006)
			Chu et al. (2003)
			Bogachek et al. (2010)
Human immunodeficiency virus-1 (HIV-1)	Cytoskeleton regulated mechanical force	Cytoskeleton rearrangement and dynamic changes leading to extensive cell mechanic changes and affecting viral replication; caveolin-1 binding to HIV Env protein and blocking viral fusion and reduced virus replication	Spear et al. (2014)
			Wang et al. (2010)
Influenza virus (IAV)	Actin and microtubule motors regulated contractile force; Shear stress; Tensile or compressive forces	Dynactin, dynein, and myosin II generating forces to help to break apart viral capsids; FAK being hijacked to promote viral replication and inhibited from activating innate immune responses; Maintaining reconstructed structural unit on a chip	Banerjee et al. (2014)
			Bergmann and Elbahesh (2019)
			Zhang et al. (2021)
Coxsackievirus B1 (CVB1)	Shear stress; Tensile or compressive forces	Maintaining reconstructed structural unit on a chip and CVB1 polarized infection	Villenave et al. (2017)
Rhinovirus (HRV)	Shear stress; Tensile or compressive forces	Maintaining reconstructed structural unit on a chip	Benam et al. (2016)
Hepatitis B virus (HBV)	Shear stress; Tensile or compressive forces	Maintaining reconstructed structural unit on a chip and achieving HBV infection *in vitro*	Kang et al. (2015), Kang et al. (2017)
			Ortega-Prieto et al. (2018), Ortega-Prieto et al. (2019)
Pseudorabies virus (PRV)	Shear stress; Tensile or compressive forces	Maintaining reconstructed structural unit on a chip	Wang et al. (2019b)

### Mechanosensors

Mechanosensors mentioned above are cellular elements that are composed of diverse proteins. However, many other mechanosensors are proteins that function individually to sense mechanical signals. For example, Notch-1 was able to response to shear stress, and was necessary for the maintenance of many cell structures and activities such as junction integrity, cell elongation, and proliferation (Mack et al., 2017). These mechanosensors are also involved in viral infection. The N-terminal portion of Notch-1 interacted specifically with the p50 subunit and inhibited p50 DNA binding of NF-κB (Wang et al., 2001). Nevertheless, like the mechanosensors discussed above, few of their effects on viral infections are studied from a mechanobiological perspective, which might be a potential study direction. One example is that Yes-Associated Protein (YAP) could suppress T-cell proliferation in a stiffness-dependent manner and regulate T-cell responses against viral infections (Meng et al., 2020). YAP functions as a mechanosensor bridging cell mechanics and viral infection. Studies focusing on mechanosensors and viral infections may elucidate how cell and tissue mechanics regulate a viral life cycle and potentially provide new antiviral targets. It is also possible to link some diseases that are closely associated with tissue mechanical changes, for example, hypertension, with viral infection, which might be able to explain why some virus infections could cause these diseases (Cool et al., 2011) and why people who suffered from these diseases had higher susceptibilities to certain viruses than healthy people (Azar et al., 2020).

A number of studies about viral–host interactions focus on regulation of signaling pathways and molecular interactions, while the underlying mechanics remain undiscussed. It is important to introduce mechanical perspectives to the study of viral–host interactions, especially in the studies of the association between viruses and structures or proteins which function as mechanosensors, since mechanical factors from both the intracellular and extracellular environment often profoundly affect the expression and function of mechanosensors, and may further regulate viral infection. Several studies on the cytoskeleton and viral infection have included views of mechanobiology, while most studies on mechanosensors and viruses lack analysis on mechanical effects of the mechanosensors on viral infection, which requires in-depth research in the future.

### Infection-Caused Cell Mechanical Changes

Viral infections can not only be regulated by cell mechanics but also cause changes of cell mechanics. A common infection-caused cell mechanical change is cytoskeleton rearrangement and dynamics, which subsequently leads to the alteration of downstream signaling pathways and variation in fundamental cell properties, such as cell stiffness, cell motility, and susceptibility to viral infections. For example, lymphocytic choriomeningitis virus (LCMV) utilized actin filaments to impel the virus to neighboring cells. Moreover, it might force infected cells to migrate faster to approach the nearest cell (Labudová et al., 2018); HIV infection changed the cytoskeleton composition of the glomerular podocyte and resulted in differed cellular stiffness (Tandon et al., 2007). JEV (Kalia et al., 2013), KSHV (Greene and Gao, 2009), Moloney murine leukemia virus (M-MLV), HIV (Gladnikoff et al., 2009), VACV (Horsington et al., 2013), and PHEV (Lv et al., 2018) infections caused actin filament rearrangements in multiple ways, which in turn facilitated their infection. Vimentin rearrangements also occurred in many viral infections like the parvovirus minute virus of mice (MVM) (Fay and Panté, 2013), enterovirus group B virus (Turkki et al., 2019), and African swine fever virus (ASFV) (Stefanovic et al., 2005).

In addition, viral infection may lead to disruption of cell–cell junctions due to their barrier function against viral infection. Viruses like RSV, human rhinovirus (HRV), influenza virus, and corona virus were able to disrupt tight junctions by targeting several tight junction proteins to facilitate their infection (Linfield et al., 2021). Adenovirus fiber protein bounded CAR and disrupted tight junction’s integrity, facilitating a virus apical escape (Walters et al., 2002). Ebola virus stimulated the Rho/ROCK pathway and then induced actin bundles formation, which generated a tensile force which loosened the VE-cadherin–formed intercellular junctions (Junaid et al., 2020). As mentioned previously, cell–cell junctions are indispensable for tissue mechanics. Therefore, disruption of cell–cell junctions by viral infection may lead to mechanical changes at the tissue scale.

Together, viral infection and cell mechanic changes are interacting complicatedly with each other. Infection-caused changes of cell mechanics may in turn generate different kinds of effects on different stages of the viral life cycle.

### Organ-on-a-Chip

The organ-on-a-chip provides a practical platform investigating host–pathogen interactions in visual microsystems. In contrast to traditional planar culturing models and animal models, these devices show exceptional advantages including convenient, low volume, low cost, and visibility. Based on available microfluidic tools, there are many unexpected findings that cannot be observed by other virus models. For example, release of CVB1 virions and inflammatory cytokines were polarized in the intestinal epithelium in a gut chip (Villenave et al., 2017). Cytokine M-CSF has been identified as a candidate marker indicating COPD caused by respiratory viruses in a lung chip (Benam et al., 2016). Moreover, organ-on-a-chip is assumed to be an optimized method to reveal novel findings about where the virus prefers to enter into or egress from and related secretory signals during infection. However, these findings are more related to phenomena without revealing detailed mechanisms. Following works will aim to figure out how mechanical forces influence biological behaviors, what is the molecular mechanism during virus infection, and which signaling pathways or mechanosensors act as a dominant role regulating mechanobiological responses. In addition, from the mechanobiological standpoint, there are still new landscapes waiting to be discovered. Owing to sophisticated interplays between mechanical forces and cells during virus infection, microsystems with more bionic structures need to be developed to better understand the mechanisms of mechanobiology in virology. Importantly, the design of an organ-on-a-chip not only requires different types of coculture cells and mechanical forces that exist in the physiological microenvironment but also resembles the authentic physiological tissue structure. In this way, experimental findings *in vitro* could potentiate the real changes *in vivo* and provide practical guidance.

On the other hand, *in silico* models, combining biology approaches with computational quantitative methods (Piñero et al., 2018), provide convenience for the analysis of a mechanical process during viral infection. When measuring forces between biomolecules directly is difficult or even impossible, *in silico* models enable people to study mechanics in host–viral interactions. By modeling the SARS-CoV-2 spike, it was revealed that the binding of the spike protein model and the host cell–surface receptor glucose regulated protein 78 (GRP78) was more favorable between regions III (C391-C525) and IV (C480-C488) (Ibrahim et al., 2020). Modeling the SARS-CoV-2 S/ACE2 complex using *in silico* approaches helped to determine the kinetic parameters of the S/ACE2 association and dissociation steps, which was essential for further *in vitro* experiments on S/ACE2-mediated viral infection (Lapaillerie et al., 2021). Moreover, the inhalation process in two medical imaging–based airway reconstructions has been established by computational fluid mechanics modeling. This demonstrated the conjecture that the nasopharynx served as the seeding region for the contamination of the lower airway *via* aspiration of SARS-CoV-2–laden boluses of nasopharyngeal fluids (Basu and Chakravarty, 2020). It is noted that a few previous studies associating virus infection with *in silico* models have been reported and summarized (Daun and Clermont, 2007; Vaziri and Gopinath, 2008; Ciupe and Heffernan, 2017; Vimalajeewa et al., 2020). It is not hard to see that there is still a long way to go to unravel the mysteries of biomechanics in virus infection by *in silico* models. Together, it will be critical to study virus infections from a mechanobiology perspective.
